# Optical characterization of colloidal CdSe quantum dots in endothelial progenitor cells

**DOI:** 10.1186/1477-3155-8-2

**Published:** 2010-02-04

**Authors:** Mátyás Molnár, Ying Fu, Peter Friberg, Yun Chen

**Affiliations:** 1 Department of Theoretical Chemistry, School of Biotechnology, Royal Institute of Technology, S-106 91 Stockholm, Sweden; 2 Department of Molecular and Clinical Medicine/Clinical Physiology, Wallenberg Laboratory, The Sahlgrenska Academy, Gothenburg, Sweden; 3 University Hospital, University of Gothenburg, SE 41345 Gothenburg, Sweden

## Abstract

We have quantitatively analyzed the confocal spectra of colloidal quantum dots (QDs) in rat endothelial progenitor cells (EPCs) by using Leica TCS SP5 Confocal Microscopy System. Comparison of the confocal spectra of QDs located inside and outside EPCs revealed that the interaction between the QDs and EPCs effectively reduces the radius of the exciton confinement inside the QDs so that the excitonic energy increases and the QD fluorescence peak blueshifts. Furthermore, the EPC environment surrounding the QDs shields the QDs so that the excitation of the QDs inside the cells is relatively weak, whereas the QDs outside the cells can be highly excited. At high excitations, the occupation of the ground excitonic state in the QD outside the cells becomes saturated and high-energy states excited, resulting in a large relaxation energy and a broad fluorescence peak. This permits, in concept, to use QD biomarkers to monitor EPCs by characterizing QD fluorescence spectra.

## Background

The use of colloidal quantum dots (QDs) is one of the most exciting developments in nanobiotechnology. Because of their high durability and unique optical properties QDs are widely used as fluorescent labelling agents for *in vitro *and *in vivo *bioimagings, such as cellular labeling, deep tissue imaging, and fluorescent resonance energy transfer donors [1]. Surface modified and water-soluble QDs open a new era in cell imaging and bio targeting as transport vehicles for therapeutic drug delivery to different diseases such as cancer and atherosclerosis [2-5].

Endothelial progenitor cells (EPCs) are heterogeneous groups of endothelial cell precursors which are circulating in the blood vessel. These cells play an important role in atherogenesis and cardiovascular regeneration [6-9]. One of the important challenges in cardiovascular research is to develop a sensitive tool that allows non-invasive in vivo tracking of EPCs, which can provide important information about site specific EPCs incorporation throughout the vasculature and whether the stage of disease alters the way EPCs are targeted.

In this work we carefully characterized confocal microscopic spectra of QDs after uptaken by EPCs. The main aim of this work is to find quantitative indicators about the interaction between the QDs and the EPCs so that we can rely on these indicators to characterize chemical and physical interactions between QDs and EPCs for *in vivo *tracking of EPCs.

## Materials and methods

**Figure 1:**
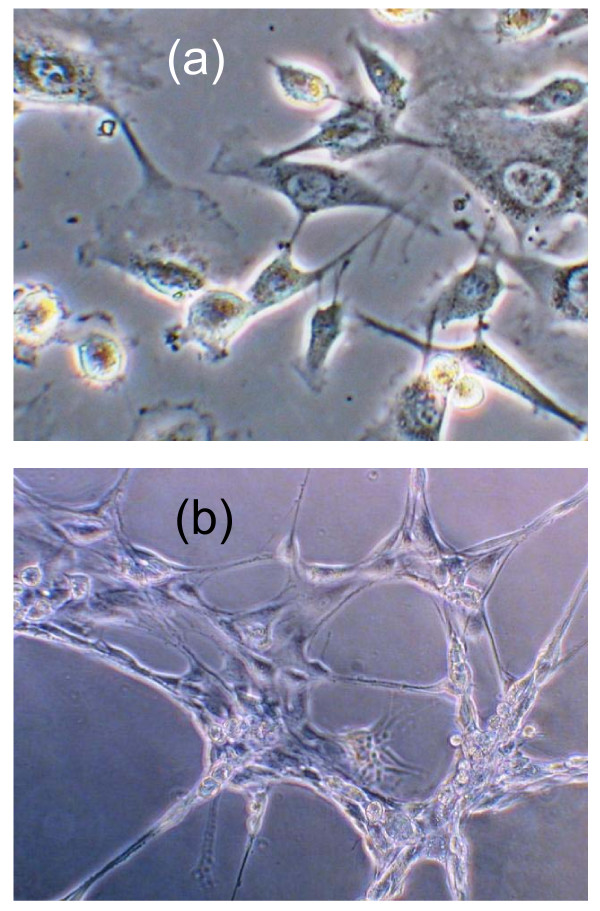
**After 7 days incubation, rat EPCs display endothelial cell-like cobblestone morphology (a) and form capillary-like structure on Matrigel (b)**.

Rat peripheral blood derived EPCs were obtained by isolating peripheral blood monocytes and incubating monocytes in endothelial cell basal medium supplied with SingleQuots (Lonza, Denmark), 10% fetal bovine serum, penicillin/streptomycin/glutamine, and 0.25 *μ*g/mL amphotericin B (Invitrogen, Sweden). After 7 days incubation, the EPCs were identified by their endothelial cell-like cobblestone morphology [Fig. 1(a)] and their ability to form capillary-like structure [Fig. 1(b)] on Matrigel (BD Bioscience, Sweden). The EPCs were then detached with trypsin, plated on a glass-bottom dish (MatTek Corporation, USA) and incubated in the cell culture medium for two days before QD labeling.

Colloidal CdSe QDs with one monolayer CdS shell (the external CdS shell was introduced for COOH derivatization), with a nominal diameter 5.5 nm and an emission wavelength of 625 nm (denoted as QD625), were chemically synthesised following the common standard method (see detailed description in Ref. [10] and references therein), which were octadecylamine coated so that they were not water soluble. They were dissolved in chloroform and a same volume of a water solution containing 3-mercaptopropionic acid (3-MPA) (1 mol/L = M) was then added under vigorous stirring for 2 hours after which QDs become water soluble. After resting the mixture for a while, chloroform and water were separated and the aqueous layer, which contained mercapto-coated QDs, was extracted. After centrifugation and decantation with water twice, an aqueous Na_2_CO_3 _solution was added to form a clear solution which was washed to remove residual 3-MPA ligands. Successive re-dispersion of QDs into water at pH 10.8 yielded a clear solution containing water-soluble QDs coated with carboxyl groups. Similar QDs were purchased from Invitrogen. Same optical characterizations were obtained using our QDs and the ones from Invitrogen so that in the following presentation we do not make further distinctions between them. QD625 were diluted in the cell culture medium to a final concentration of 16 nano-M (nM) and were added to the EPCs. Here unlike conventional organic and inorganic chemicals, the concentration of colloidal QDs is difficult to determine by gravimetric methods. It is usually expressed as molar concentration determined via molar extinction coefficient measurement [11]. The cells were incubated with QD625 for 30 hours. After QD incubation the cells were washed with phosphate-buffered saline (PBS, pH 7.2), fixed with 4% paraformaldehyde for 10 minutes and then stored in PBS at 5°C for confocal microscopy measurements (note that the confocal microscopy measurements were performed at room temperature).

Leica TCS SP5 Confocal Microscopy System was used to characterize the optical properties of these samples. Images were captured with a scanning speed of 400 Hz and image resolution of 512 × 512 pixels, and then analysed using Leica Application Suite 2.02.

## Results and discussion

**Figure 2:**
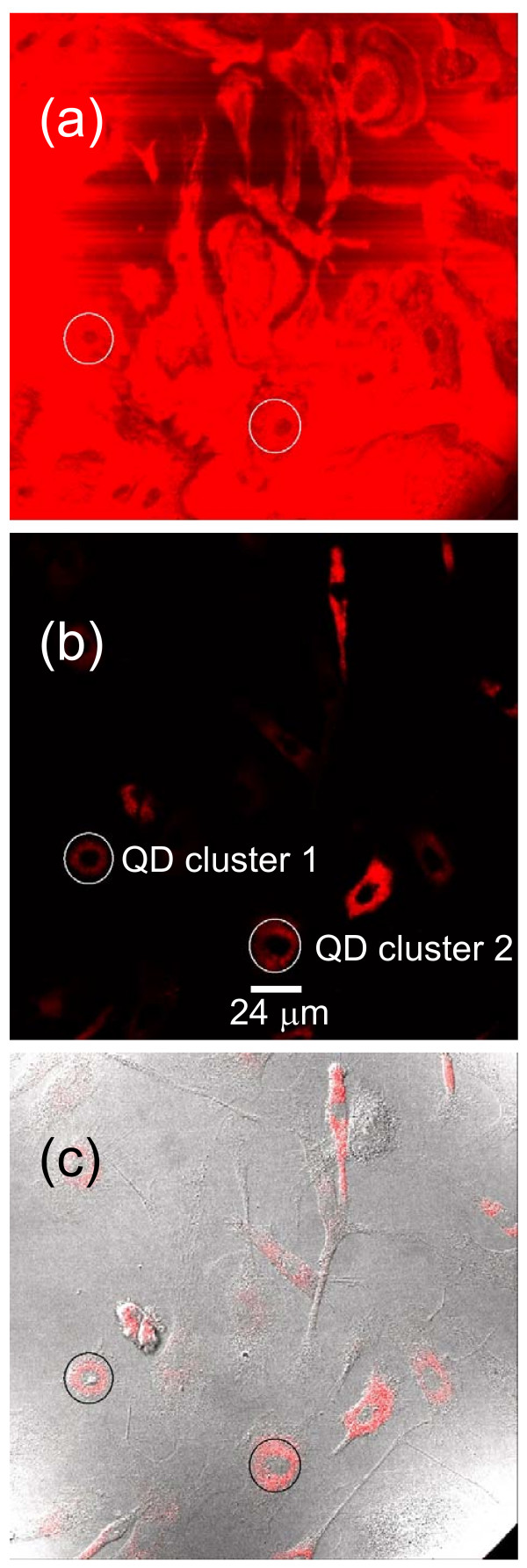
**Confocal imaging of QD-uptaken EPCs at 450 nm (a) and at 616 nm (b)**. (c) is the confocal imaging at 616 nm merged with a differential interference contrast image. Built-in excitation laser source at 458 nm was used. Excitation power control was 20%.

Fig. 2 shows typical confocal slice images using the excitation laser source at 458 nm.

**Figure 3:**
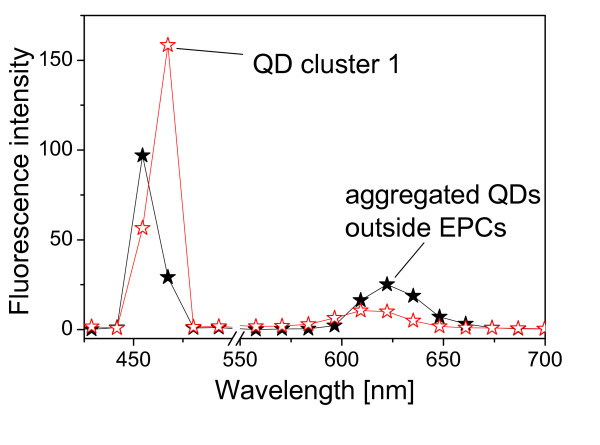
**Confocal spectra of intracellular QD1 (hollow stars) and an aggregated QD cluster located outside EPCs (solid stars)**. The wavelength of the excitation laser source is 458 nm. Excitation power control is 20%. The wavelength scanning step is 12 nm.

**Figure 4:**
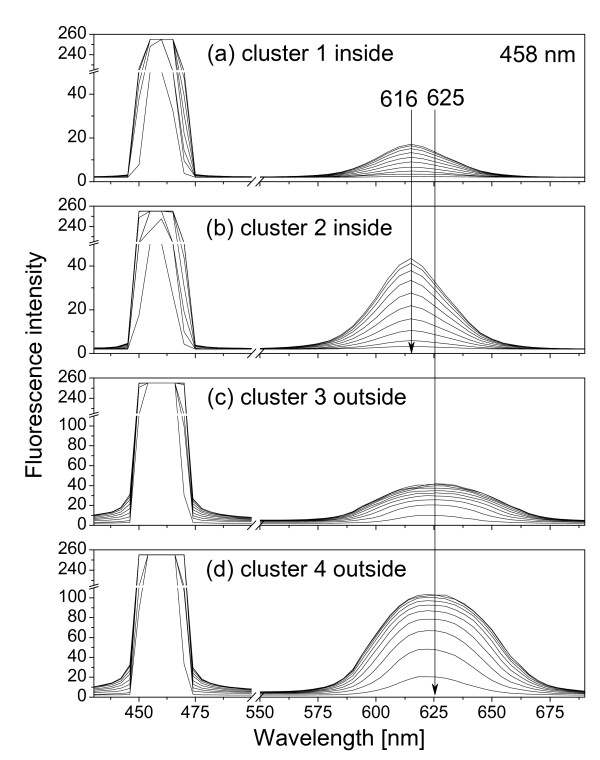
**Fluorescence spectra of QDs inside and outside cells**. Ten fluorescence spectra for each QD cluster were obtained using ten microscopy excitation powers (lowest = 10% and highest = 100%). Excitation wavelength is 458 nm. (a) QD cluster 1 inside cell, (b) QD cluster 2 inside cell, see Fig. 2; (c) QD cluster 3 outside cells; (d) QD cluster 4 outside cells.

We analyzed optical spectra of QD clusters located inside EPCs [QD cluster 1 as marked in Fig. 2(b)] and one aggregated QD cluster located outside the EPCs (not shown). Their confocal spectra are shown in Fig. 3. The diameter of the areas measured were 24 *μ*m in all cases. Two major effects can be observed in Fig. 3. The first one is the strong reflection of the excitation radiation from the EPCs (458 nm) from the area of QD cluster 1 [see Fig. 2(b)] as compared with the areas outside EPCs. It can be more clearly observed in Fig. 2(a) which was obtained at 450 nm. Note that the central wavelength of the excitation laser 458 nm is 458 nm and its full width at half maximum (FWHM) is about 12 nm [obtained from the 10% excitation-power spectrum, see Fig. 4(a) below]. The signals at 458 nm were already saturated when 20% excitation power was used so that Fig. 2(a) is shown at 450 nm (i.e., at the edge of the excitation peak which is centred at 458 nm with a FWHM of about 12 nm) in order to be able to show the spatial structure of the sample. Strong reflection indicates less transmission and thus less excitation so that the ratios between the excitation radiation signal and the QD fluorescence are different for QD cluster 1 and QD clusters outside EPCs. Another important effect is the blue-shift of the fluorescence from QD cluster 1 inside the EPC (616 nm with respect to 625 nm outside EPCs).

Note that the cells were washed after QD incubation so that QDs are not expected to remain outside cells in the sample. However, we occasionally observed QD clusters stuck to small particles. These particles remained loosely attached to the bottom of the dish after washing steps. The optical spectrum of one of such QD cluster outside EPCs is shown in Fig. 3 measured by using a wavelength scanning step of 12 nm. It was difficult to measure confocal spectra at smaller scanning steps since these loosely attached QDs were moving. In order to be able to do precise quantitative comparison, we prepared reference QD samples (QD cluster 3 and cluster 4) simply by drying one drop of 8 *μ*M carboxyl-coated QD625 solution on a glass-bottomed dish so that QDs are not mobile.

**Figure 5:**
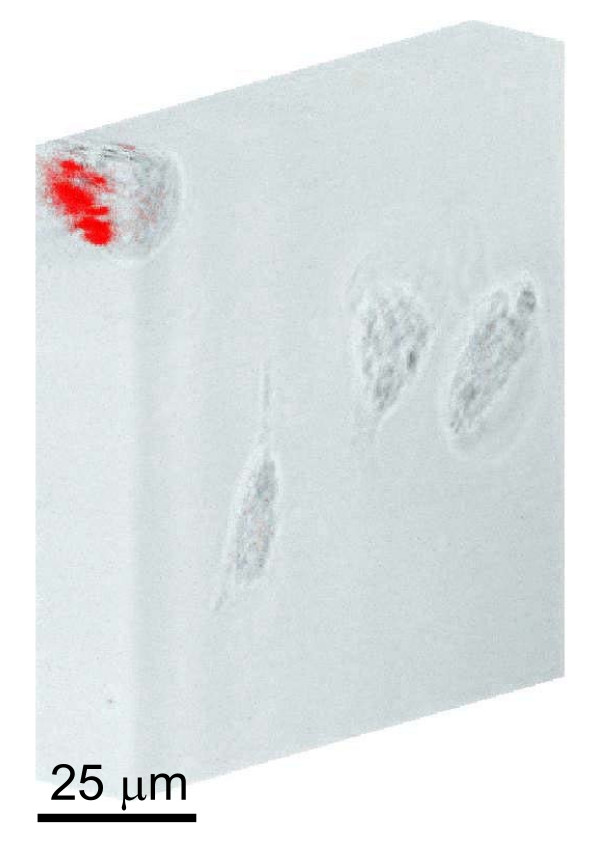
**Three-dimensional confocal imaging at 616 nm**. A cross section of an endothelial progenitor cell is shown in the upper left corner. QDs (red) are located in the middle of the EPC surrounded by the cell membrane.

**Figure 6:**
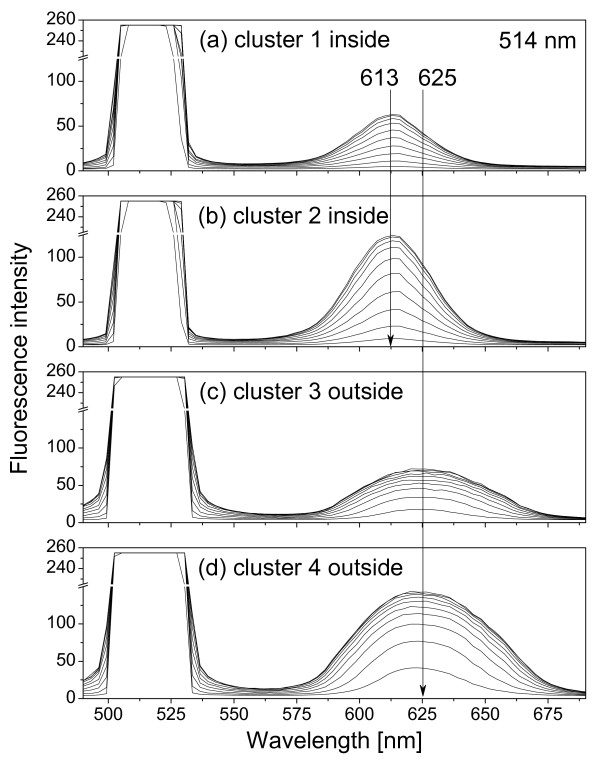
**Same as Fig. 4 except the excitation wavelength is 514 nm**.

Fig. 4(a-b) show the confocal spectra of clusters 1 and 2 in Fig. 2, together with those of clusters 3 and 4, measured using ten different excitation power settings (lowest = 10% and highest = 100%). To ensure the intracellular localization of QDs, a series of 163 sequential images that covers the whole cell volume (from the top down to the bottom, total thickness 19.6 *μ*m) were acquired by the confocal microscope from which three-dimensional image (Fig. 5) was re-constructed showing that QDs (red) did locate in the middle of the cell surrounded by the cell membrane. Note furthermore that EPCs under investigation were disk like with a breadth of about 20 *μ*m and a thickness of about 20 *μ*m, see Fig. 5. Fig. 6 shows the fluorescence spectra similar to Fig. 4 but obtained by using the built-in excitation laser at 514 nm.

Note the difference in fluorescent emission peaks (616 nm and 613 nm) when 458 nm and 514 nm LASER wavelengths are used for cluster 1 and 2 in Figs. 4(a-b) and 6(a-b). The most probable reason is the merging of the laser signal with the QD fluorescent signal when the 514 nm laser is used, especially at high excitation powers. The fitted wavelength of the peak at about 613 nm in Figs. 6(a-b) actually blue shifts from 615 to 613 nm following the increase of the 514-nm laser power.

**Figure 7:**
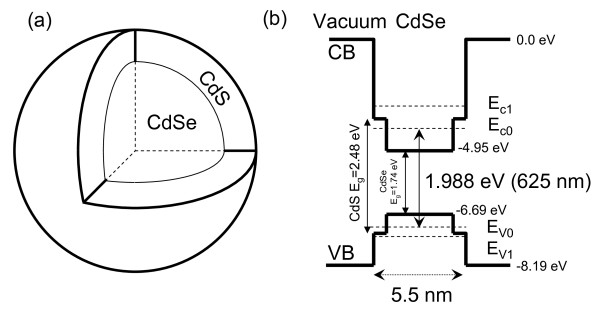
**(a) Geometric structure of the CdSe QD with one monolayer CdS shell**. (b) Schematic energy band structure of the CdSe QD.

**Figure 8:**
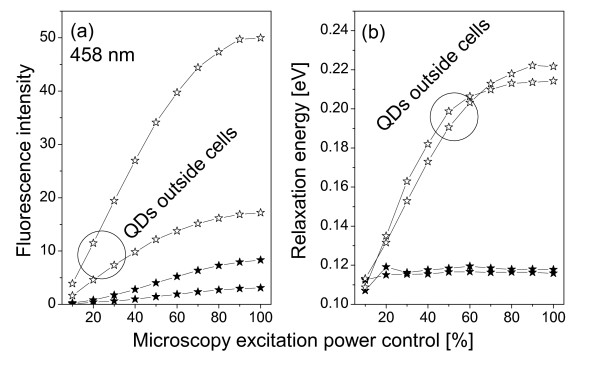
**Confocal spectral characterizations of QDs**. Excitation wavelength is 458 nm. (a) Fluorescence intensity; (b) Relaxation energy. Solid stars: QDs inside cells; hollow stars: QDs outside cells (curves are grouped by circle).

**Figure 9:**
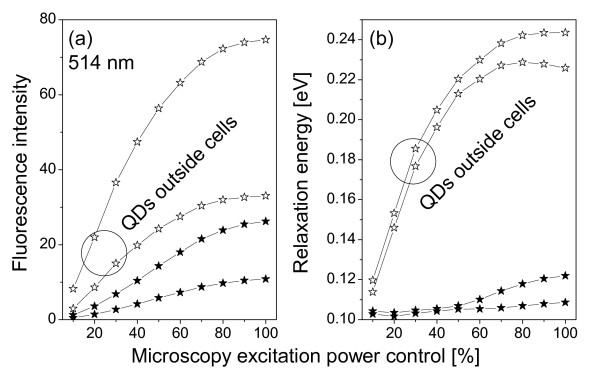
**Same as Figs. 8 but excitation wavelength is 514 nm**.

The energy band structure of the CdSe QD is schematically shown in Fig. 7, where CB denotes the conduction band edge and VB the valence band edge. Referring to the vacuum level as potential energy zero, the CB of CdSe is -4.95 eV (electron affinity of CdSe), the band gap *E*_*g *_(energy difference between CB and VB) is 1.74 eV, and the quantum confinement energy for the valence band hole is 1.5 eV [12]. For our CdSe QDs with a diameter of 5.5 nm (including the one monolayer CdS shell), the energy separation between the ground electron state, i.e., *E*_*c*0 _in Fig. 7, in the conduction band and the ground hole state (*E*_*v*0_) in the valence band is 1.988 eV, corresponding to the emission wavelength of 625 nm. Because of the quantum confinement effects in QDs, electron states in the conduction band (hole states in the valence band) become quantized as *E*_*c*0_, *E*_*c*1 _etc (*E*_*v*0_, *E*_*v*1 _etc), where *E*_*c*0 _and *E*_*v*0 _denote the ground electron and hole state, respectively. QD fluorescence due to the recombination of electron at *E*_*c*0 _and hole at *E*_*v*0 _is described by a Lorentzian peak [13](1)

where *ħω *is the photon energy, *ħω*_0 _= *E*_*c*0 _- *E*_*v*0 _is the excitonic energy in the QD, Γ is the relaxation energy, *A *the fluorescence intensity. The values of these fitting parameters for spectra in Figs. 4 and 6 are shown in Figs. 8 and 9.

In the course of this work two major effects were observed. First is the blue shift of QD fluorescence peak following their uptake by the EPCs. It has been shown that QDs with carboxylic acid surface coatings were recognized by lipid rafts in human epidermal keratinocytes and internalised into early endosomes then transferred to late endosomes or lysosomes [14]. For our QDs inside EPCs shown in Fig. 5, the most probably modifications to the quantum confinement of electrons and holes in the CdSe QD are interactions between surface atoms and lipids and proteins (mostly interacting with Cd atoms) as well as ions such as K^+ ^(mostly interacting with S atoms) inside the cell so that the covalent electrons of the surface Cd and S atoms are no longer in the energy band structure of Fig. 7. The effective radius of quantum confinement is reduced for the exciton inside the QD and the excitonic energy becomes increased. As was shown [15](2)

where *E *= *ħω*_0 _is the excitonic energy, *r *is the QD radius, *E*_*g *_is the energy bandgap of the QD material, *δr *and *δE *are modifications in radius and excitonic energy. For our CdSe QD625, the nominal diameter is 5.5 nm. Assuming one monolayer modification (about 0.3 nm [12]) in the radius, Eq. (2) gives us *δE *= 30 meV, which agrees very well with Figs. 8 and 9. Note that the fitted fluorescence peak position for 458 nm excitation is different from the 514 nm excitation, 616 nm vs 613 nm in Figs. 4 and 6, which we believe is due to the mixtures between the excitation signal and the QD fluorescence. For 514 nm excitation, the mixture is stronger so that the blue shift appeared to be larger.

Zhang et al. reported similar blue shift of fluorescence peak of thiol-capped CdTe QDs within less than 10 min of QD uptaking in living cells caused by surface photooxidation [16]. The reported blue shift in CdTe QDs is much larger than our cases. Furthermore, the peak width of CdTe QDs is largely increased, while it remains basically unchanged for our CdSe QDs. The major differences between CdTe QDs and our CdSe QDs are probably due to the fact that the oxidation of Te atoms are relatively easy, therefore CdTe QDs are less chemically stable.

The other important finding is that the relaxation energy in the QDs inside cells is relatively small and independent of the excitation power, while it increases quickly in the QDs outside of cells then saturates as a function of the excitation power, see Figs. 8(b) and 9(b). The large relaxation energy is actually an indication of the saturation of the ground excitonic state occupation and the occupations of high-energy excitonic states due to the large optical pumping by the excitation radiation.

The same effects (blue shift and the relaxation energy behavior) were obtained for QD625 (emission wavelength 625 nm) under the excitations of 458 and 514 nm wavelengths. The insensitivity to the excitation wavelength can be theoretically expected when the excitation energy is not too high compared with the excitonic energy of QDs (i.e., in the range of one-photon and multiphoton excitations) [17]. High energy radiation (larger than twice the excitonic energy) was shown to induce multicarrier excitation [18] so that it may induce different characterizations in the QD fluorescence spectrum.

Similar measurements were repeated two and four months late on randomly chosen QD clusters, and we found that both the samples and measurement results were very stable when the same measurement setups were used. We noticed that as long as measurement performances are careful, there are no significant changes in the confocal spectral characteristics (i.e., the fluorescence intensity, excitonic energy and relaxation energy).

## Conclusions

We have shown that the uptaking of colloidal QDs by EPCs effectively reduces the radius of the exciton confinement inside the QDs so that the excitonic energy increases and the peak of the QD fluorescence blue shifts. Furthermore, the cell environment surrounding the QDs shields the QDs so that the excitation of the QDs inside the cells is usually weaker. QDs outside the cells are excited to higher degree, which leads to the saturation of the ground excitonic state. The excitation of high-energy states results in a broader fluorescence peak.

Our study shown that intracellular environment can affect optical characteristics of QDs and that such changes are quantifiable. Therefore, changes of QD fluorescence spectra should allow one to characterize the interaction between colloidal QDs and EPCs. This should facilitate the development of QD biomarkers for monitoring EPCs at sub-cellular level.

## Competing interests

The authors declare that they have no competing interests.

## Authors' contributions

All authors contributed equally, read and approved the final manuscript.
